# Five years of the National Rural Health Mission – what lessons for policy?

**DOI:** 10.1186/1753-6561-6-S1-I1

**Published:** 2012-01-16

**Authors:** Narayanan Devadasan, NS Prashanth

**Affiliations:** 1 Institute of Public Health, Bangalore, India

## 

In the late 1800s, Mark Twain wrote, “There is only one India...a wonderland of fabulous wealth and fabulous poverty, of splendour and rags. The one sole country under the sun that is endowed with an imperishable interest for alien princes and peasants, for lettered and ignorant, wise and fool, rich and poor...” [1].

A 100 years later and we couldn't agree more. India is a land of massive inequity. While urban women are treated to specialised and luxurious maternity clinics that promise to be “a home away from home” - rural women are dying in their real homes, unable to get to the nearest health centre, which isn't so near after all. But there is a silver lining to this. A lot of reforms have taken place in the health sector - reforms, that promise a great deal of change. However, there is very little empirical evidence to back this change and that poses a major hurdle to effective evaluation and course correction. There is little or negligible evidence from the field about what is working and what isn't. And importantly, why it is working and why it isn't.

As we draw close to the release of the 2011 World Health Report for which the chosen theme is “No health without research” [2], we are faced with limitations of research capacity and outputs [3]. Despite several calls for more implementation research [4] to guide health policies, the lack of right forum for policy makers and researchers to discuss relevance of the research topic limits the implementation of such recommendations. And even if there is some evidence in the form of research or studies, they are too few and too far apart. And these few studies are not disseminated widely enough and/or to people that matter in bringing about a change.

So the situation we are confronted with is this: on one hand, researchers who have studies – crucial evidence from the field – that are highly academic and circulated within a very close milieu and often not tailored to policy and needs of practitioners. On the other hand, decision makers/policy makers who are forced to make decisions in a vacuum, based on strong convictions of policy actors, impressions or conventions. Strong calls for universal health coverage such as the recent one by Reddy et al. [5] have had to depend largely on experiences and convictions rather than being informed by strong evidence from implementation research from the field.

In response to this glaring gap, we conceptualised a national conference to bring together researchers, practitioners-managers and policymakers to bridge this gap between research and policy and give both of these, very important groups a chance to interact. The idea of the conference is to give them a common platform for effective dialogue so that researchers can share their findings with key decision makers and policy makers can in turn, evaluate the policies presently in practice. Not to mention, introduce and implement newer policies that are backed by evidence.

The conference focuses on the National Rural Health Mission (NRHM) that was launched by the Indian government on 12^th^ April 2005. NRHM was launched to provide accessible, affordable, accountable, effective and reliable health care facilities to rural areas in India through increased financial outlays, flexible financing mechanisms, increased community involvement, horizontal integration of programmes, improved management at district and sub-district levels through capacity-building and innovative human resource management [6]. NRHM was launched on a “mission” mode recognising the urgency and importance of improving the status of health care provision in India.

As the NRHM completes five years, the Institute of Public Health felt that it was a good idea to look back on what has been achieved till date. Ignoring the myriad exaggerated and subjective criticism it has, and still receives, there is a need to question if there is empirical evidence that the NRHM is actually making a difference?

The conference stands as an opportunity for researchers to present the results of their empirical studies on health systems on an international dais.

In this supplement, we have put together 24 papers that were presented at the first National Conference on Bringing Evidence into Public Health Policy (EPHP) held in Bangalore during 10-11 December 2011. By presenting these findings to policy makers and implementers at both, the central and state levels, there is a hope to influence the policy making process through evidence based research. For policy makers, it is a chance to get together, review the evidence and act accordingly. Through the conference, the researchers would get a chance to actively participate in the policy and program development process and distil crucial research questions in the future. Given the power of research, such a platform poses as an impetus, inspiring other researchers to take up health systems research and subsequently, with a union of research and action, strengthen the existing health system.
